# Dexmedetomidine attenuates high-fat diet-induced metabolic dysfunction-associated steatotic liver disease in rats by regulating the autophagic pathway

**DOI:** 10.1038/s41598-026-38250-y

**Published:** 2026-03-13

**Authors:** Sally M. Ezzat, Maha H. Sharawy, Ghada M. Suddek

**Affiliations:** Department of Pharmacology and Toxicology, Faculty of Pharmacy, Mansoura University, Mansoura, 35516 Egypt

**Keywords:** Apoptosis, Autophagy, Dexmedetomidine, High-fat diet (HFD), Metabolic dysfunction-associated steatotic liver disease (MASLD), Autophagy, Pharmacology, Non-alcoholic fatty liver disease, Non-alcoholic steatohepatitis

## Abstract

Steatosis is the accumulation of neutral lipids in the cytoplasm. Metabolic dysfunction-associated steatotic liver disease (MASLD) is a suitable acronym to define steatosis associated with metabolic dysfunction. Autophagy is a conserved quality-control process in lysosomes that destroys cytoplasmic contents. Lipophagy is the process through which autophagy destroys lipid droplets. This study investigates effects of dexmedetomidine (Dex) on MASLD induced by high-fat diet (HFD) in rats. Sprague Dawley rats were fed 60% HFD for 8 weeks, and Dex (1 mcg/kg/day and 5 mcg/kg/day) was given intraperitoneally. Dex decreased liver integrity markers and improved antioxidant status by decreasing malondialdehyde (MDA). In addition, it decreased serum levels of both total cholesterol and triglycerides. Autophagic markers (Beclin 1, ULK1, AMPK, LC3) were improved by Dex treatment and protein expression of p62 was decreased in a dose-dependent manner. Finally, it decreased apoptosis by decreasing caspsae-3 and increasing Bcl-2. Histopathological examination was used to assess the degree of steatosis. Dex was found to induce autophagy in MASLD in a dose-dependent manner and improve liver function and degree of steatosis. Dex, therefore, may be a potential adjunctive therapeutic option in clinical settings for treating MASLD.

##  Introduction

The liver is an important site for fatty acid packaging, redistribution, and digestion. As a result, liver diseases can result in an increased accumulation of fat content, a condition known as hepatic steatosis, which predisposes individuals to far more severe consequences^1^. Fatty liver associated with metabolic dysfunction is common, affecting a quarter of the population^2^. Non-alcoholic fatty liver disease (NAFLD) is the leading cause of chronic liver disease in the industrialized world, and it is frequently associated with metabolic comorbidities such as diabetes, hypertension, dyslipidemia, and obesity^3^. In 2020, the term “metabolic dysfunction-associated fatty liver disease (MAFLD)” was proposed to substitute NAFLD, to refer to fatty liver disease caused by systemic metabolic dysregulation. Then in 2023, the most recent term metabolic dysfunction-associated steatotic liver disease (MASLD) was proposed. The “two-hit theory” is a time-tested paradigm for the development and progression of MASLD. Bad living habits also further increase blood level of fatty acids, and excessive fatty acids are transported to the liver, exceeding their transport capacity, and deposited in the liver, leading to steatosis, which is the “first blow.”^4,5^. Long-term excessive fat accumulation causes endoplasmic reticulum stress, mitochondrial dysfunction, and oxidative stress, resulting in the release of inflammatory molecules, exacerbating liver cell damage and accelerating the change of simple fatty liver to metabolic dysfunction-associated steatohepatitis (MASH), which is referred to as the “second blow.“^6,7^. First, it was postulated that lipophagy protects against the first hit by preventing excessive fat formation, Second, autophagy prevents the second hit by removing damaged reactive oxygen species-generating mitochondria while maintaining hepatocellular ATP levels^8^.

Autophagy is an important mechanism that regulates liver metabolism, not only as a nutrient stress rescue pathway, but also in physiological responses to the fasting-feeding cycle^9^. It is a conserved quality-control process in lysosomes that destroys cytoplasmic contents. Dysregulation of autophagy has been seen in a variety of clinical states, including obesity and type 2 diabetes, inflammatory and viral disorders, neurodegenerative diseases, and cancer^10,11^. In mammalian cells, the term autophagy refers to three processes: macroautophagy, microautophagy (including endosomal microautophagy), and chaperone-mediated autophagy^12^.

In this study, we focused on autophagy and its recently elucidated function in cellular lipid utilization, termed lipophagy^8^. Lipophagy is the process through which autophagy destroys lipid droplets (LD)^13^. LDs, neutral lipid–filled organelles, are sequestered by autophagic membranes and delivered to lysosomes, where lipases break down the lipids and release free fatty acids (FFAs) for mitochondrial β-oxidation or other metabolic uses^13^.

Light chain 3- II (LC3II), Beclin1, and p62 are among key autophagic markers^14^. In autophagy, LC3II serves as a hallmark of autophagosome formation and maturation and is used widely as a marker of autophagic flux^15^. Beclin1 is an evolutionarily conserved protein that is recognized as a positive regulator of autophagy; p62 is a multi-domain adaptor protein and a key player in autophagy-dependent quality control^14^. A previous study showed that AMP- activated protein kinase (AMPK) directly phosphorylates unc-51 like autophagy-activating kinase 1 (ULK1), which is required for selective autophagy^16^.

Dexmedetomidine (Dex), a selective α_2_ adrenoceptor agonist, not only has sedative and anxiolytic effects, but also has sympathetic nerve suppression and anti-inflammatory activities. As a result, it is widely used in clinical anesthesia and the intensive care unit^17^. Mounting experimental studies have demonstrated that Dex protects against various organ injuries using different animal models due to the anti-inflammatory action and immunoregulatory effects^18,19^.

In steatosis/MASLD models, Dex showed promising hepatoprotective role in body metabolic homeostasis. In a mouse model of HFD, DEX reduced steatosis, inflammation and improved insulin resistance and lipophagy markers^20^. Furthermore, it was proved that Dex induced autophagy in septic liver injury by upregulating the sirtuin1/adenosine monophosphate-activated protein kinase signaling pathway, that is an important survival signaling mechanism involved in autophagy modulation. It also increased the expression of Beclin-1, LC3II and decreased expression of p62^18^.

Regarding the safety of DEX in steatotic liver, a previous clinical study stated that using the drug in dose per kilogram of total body weight, was not appropriate for the obese patients, as it resulted in higher plasma concentrations than those observed in lean subjects. This may be attributed to the compromised blood flow to the liver in case of obesity and steatosis^21^, however, when doses were adjusted to lean body weight, no negative influence of obesity in Dex clearance was observed. Making the use of lean body weight as a scaler, for obese patients is the ideal strategy to prevent Dex accumulation^22^.

In the present study, our objective was to investigate the effect of Dex on MASLD and highlight the role of autophagic pathway in its protective effects.

## Materials and methods

### Animals

Thirty-two adult male Sprague Dawley rats with an average age of 6–8 weeks and weighing 150 ± 20 g were used. The rats were purchased from the Egyptian Organization for Biological Products and Vaccines (VACSERA, Giza, Egypt). Four animals were housed in each cage at constant environmental conditions throughout the experimental period at room temperature 25 °C ± 2 with a 12-h light/dark cycle.

The protocol was accepted by the institutional Research Ethics Committee of the Faculty of Pharmacy at Mansoura University in Egypt, code number: (2021-224/2023-231), which is in accordance with Protocol of Laboratory Animal Care (NIH publication no. 85 -23, amended 1985). All methods were performed in accordance with the ARRIVE guidelines.

### Drugs and chemicals

Dex was purchased as a pharmaceutical product (Precedex vial 100 mcg/ml) from Pfizer Pharmaceuticals (NY, USA). Dex injection concentrate (100 mcg/ml) was diluted in 0.9% sodium chloride solution to achieve the required concentration prior to administration. The diluted Dex in 0.9% sodium chloride was stable for at least 48 h at 20–25 °C and 14 days at 5 °C when stored in polypropylene syringes.

### Experimental design

Rats were assigned randomly into 4 groups (8 rats/group) as follows: group 1: control group: rats received regular chow for 8 weeks; group 2: HFD group: rats received 60% HFD for 8 weeks; group 3: HFD + Dex (1 mcg/kg, i.p.)^23^: rats received 60% HFD and Dex (1 mcg/kg, i.p.) daily for 8 weeks; group 4: HFD + Dex (5 mcg/kg, i.p.)^23^: rats received 60% HFD and Dex (5 mcg/kg) daily for 8 weeks.

Regular chow was composed of 60% carbohydrates, 20% protein, 4% fats, 2% fibers, and 14% minerals and vitamins. The HFD was 60% beef tallow and 40% ordinary chow. For 8 weeks, all rats had free access to drinking water and food. The body weights were recorded on a weekly basis.

### Sample collection

At the end of the experiment, rats were fasted for 12 h and given unrestricted access to water before being anesthetized with secobarbital sodium (50 mg/kg, i.p.). Blood was taken from rats via retro-orbital puncture and centrifuged at 4000 rpm for 15 min to separate serum (to be used to assess liver function markers and lipid profile). The anesthetized animals were euthanized by cervical dislocation. Immediately following sacrifice, liver tissues were harvested and kept at − 80 °C for further assessment of oxidative stress markers (GSH and MDA using colorimetric assays) and autophagic markers (AMPK α1, ULK1, Beclin1 and LC3 using ELISA technique). Another section of the liver was fixed in 10% buffered formalin for histological examination using H&E stain and immunohistochemical investigation of autophagic marker p62, apoptotic marker caspase-3 and antiapoptotic marker Bcl-2.

### Histopathological examination of hepatic tissues

The liver tissue was fixed in 10% buffered formalin, embedded in paraffin wax, sectioned (6 μm), and stained with hematoxylin and eosin (H&E). Steatosis, inflammation, fibrosis, and ballooning degeneration were all assessed using H&E-stained liver sections. Steatosis was rated blindly by a pathologist, and the severity was categorized into the following categories depending on the percentage of total area affected: Inflammation was graded from 0 (5%), 1 (5–33%), 2 (34–66%), to 3 (> 66%). Ballooning degeneration was graded from 0 to 2, while fibrosis was graded from 0 to 3^24^.

### Assessment of liver integrity markers

The collected blood serum was used for measurement of alanine aminotransferase (ALT) activity, aspartate aminotransferase (AST) activity, gamma-glutamyl transferase (GGT) using enzymatic colorimetric kits purchased from Human (Wiesbaden, Germany).

### Measurement of lipid profile in serum

Serum triglycerides (TG) and total cholesterol (TC) were estimated using commercial kits (Spinreact, Santa Coloma, Spain).

### Evaluation of oxidative stress and antioxidant biomarkers

Oxidative stress and antioxidant markers were assessed in hepatic tissue homogenates of the various groups. Reduced glutathione (GSH) level, as a defensive antioxidant marker, was determined using colorimetric GSH Kit (Biodiagnostic, Giza, Egypt) following the reported protocol^25^. The Lipid peroxidation marker, malondialdehyde (MDA), was measured using MDA colorimetric kit (Biodiagnostic, Giza, Egypt) following the mentioned protocols^26,27^.

### Assessment of AMPK α1, ULK1, Beclin1 and LC3 by ELISA

Sandwich ELISA technique was used to measure rat AMP-activated Protein Kinase alpha 1 (AMPKα1) in cell lysates (R&D Systems, USA). Rat ULK1 (serine/threonine-protein kinase) was measured by ULK1 ELISA Kit (Mybiosource, Vancouver, Canada). Beclin1 was measured by Beclin1 ELISA kit (Novus Biologicals, LLC, USA). LC3 was measured using an ELISA kit (AFG Bioscience, USA).

### Immunohistochemical analysis of p62

For immunohistochemical analysis of p62, primary antibodies against p62 (ABclonal, USA, dilution 1:200, Cat. No. A11250) were used in accordance with the standard protocols.

### Immunohistochemical analysis of apoptotic markers caspase-3 and Bcl-2

For immunohistochemical analysis of caspase-3 and Bcl-2, primary antibodies against active caspase-3 rabbit polyclonal (dilution 1:1000, Cat. No. GB11532, Service Bio) and Bcl-2 mouse monoclonal (dilution 1:100, Cat. No., Genemed biotechnologies) were used in accordance with standard protocols.

### Statistical analysis

The results were expressed as mean ± SD. Inter-group significance was examined using one-way analysis of variance (ANOVA) followed by Tukey’s post-hoc test. Histopathological scores were analyzed using the non-parametric Kruskal-Wallis test followed by Dunn’s post-hoc test. Statistical tests were performed by GraphPad Prism v9.0.0.121 software (San Diego, California, USA). Statistical significance was considered at *p* < 0.05.

## Results

### Effect of Dex on histopathological changes in liver

**Fig. 1:**
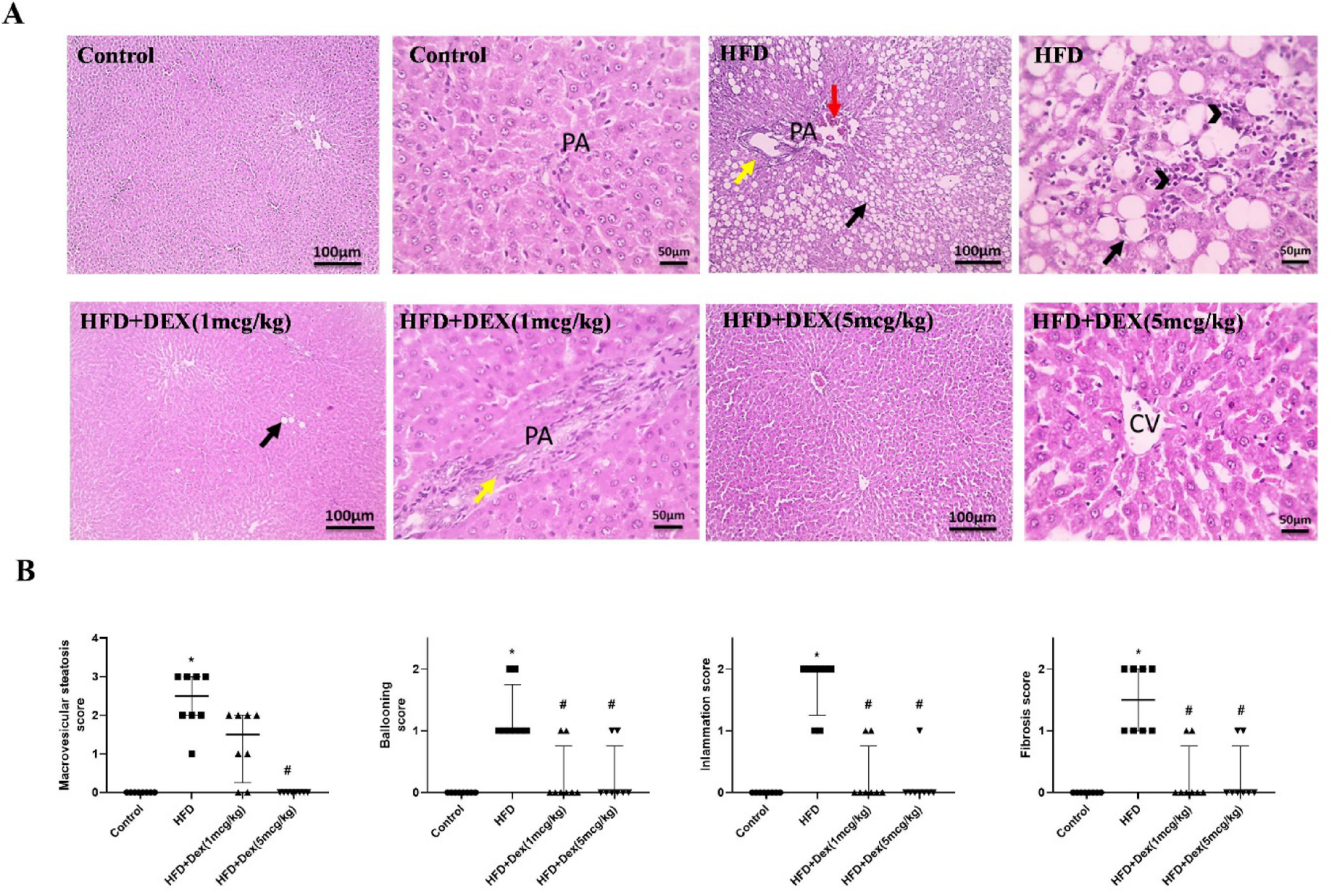
Histopathological changes in the liver. (**A**) Microscopic images of H&E-stained liver specimens from control rats showing normally organized liver parenchyma and hepatocytes, central veins (CV). Low magnification in HFD group shows diffuse macrovesicular steatosis in hepatocytes (black arrows), congested blood vessels (red arrows), in addition to mononuclear cell infiltration (arrowheads). Sinusoids are narrowed or obliterated. Sections from Dex 1 mcg/kg rats showing mild macrovesicular steatosis in a few hepatocytes (black arrows), proliferated bile ducts (yellow arrows). Sinusoids are normal. Whereas sections from Dex 5mcg/kg rats are showing a retained normal appearance of hepatocytes, CV, portal areas (PA) and sinusoids. Low magnification X: 100 and high magnification X:400. (**B**) Scoring for steatosis, ballooning, inflammation, and fibrosis. Data are represented as median ± interquartile range (*n* = 8). ^*, #^*p* < 0.05, significantly different vs. Control, HFD, respectively, Kruskal-Wallis was used followed by Dunn’s test as post hoc test.

In order to investigate the extent of liver damage and steatosis caused by HFD and the protective effects of Dex, liver architecture was examined using H&E stain. HFD group showed diffuse macrovesicular steatosis in hepatocytes, expanded portal areas (PA), congested blood vessels, dilated and proliferated bile ductules. Sinusoids are narrowed and obliterated in addition to mononuclear cell infiltration. Dex treatment counteracted these effects dose dependently. Figure 1A illustrated the histopathological alterations that affect the liver sections from all groups, and Fig. 1B showed deviations in histopathological scores.

### Effect of Dex on liver markers

**Fig. 2:**
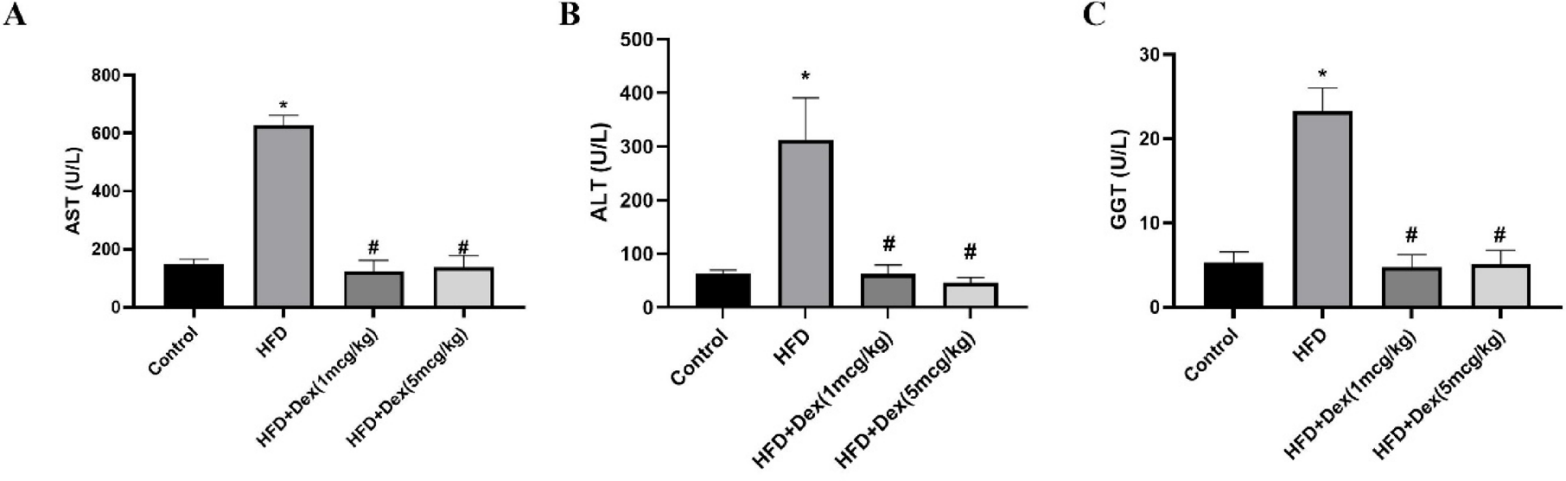
Aspartate aminotransferase (AST), alanine aminotransferase (ALT), and gamma-glutamyl transferase (GGT) levels. Sprague Dawley rats were fed 60% HFD for 8 weeks, and Dex (1mcg/kg/day and 5mcg/kg/day) was given intraperitoneally. Liver markers were assessed in serum. Panels (**A**), (**B**), and (**C**) correspond to AST, ALT, and GGT activities, respectively. Data are represented as mean ± SD (*n* = 6–8). *, # *p* < 0.05, significantly different vs. Control, HFD, respectively, using one way ANOVA followed by Tukey post hoc test.

Assessing liver integrity markers is crucial to assess liver function. In this context, liver transaminase enzymes and GGT were measured. AST, ALT and GGT levels were increased in HFD group by 4.21-fold (from 149 ± 16.2 to 627.3 ± 33.7), 4.93-fold (from 63.4 ± 6.2 to 312.6 ± 77.9) and 4.41-fold (from 5.3 ± 1.3 to 23.3 ± 2.8) fold, respectively, compared to control group. Dex (1 mcg/kg) and Dex (5 mcg/kg) resulted in significant reduction in AST levels by 80.25% (from 627.3 ± 33.7 to 123.9 ± 37.4) and 78% (from 627.3 ± 33.7 to 137.8 ± 40.1) (Fig. 2A), ALT levels by 79.9% (from 312.6 ± 77.9 to 62.6 ± 16.3) and 85.3% (from 312.6 ± 77.9 to 46 ± 10.2) (Fig. 2B) and GGT levels by 79.6% (from 23.3 ± 2.8 to 4.8 ± 1.5) and 78% (from 23.3 ± 2.8 to 5.1 ± 1.6), respectively, Fig. 2C).

### Effect of Dex on lipid profile

**Fig. 3:**
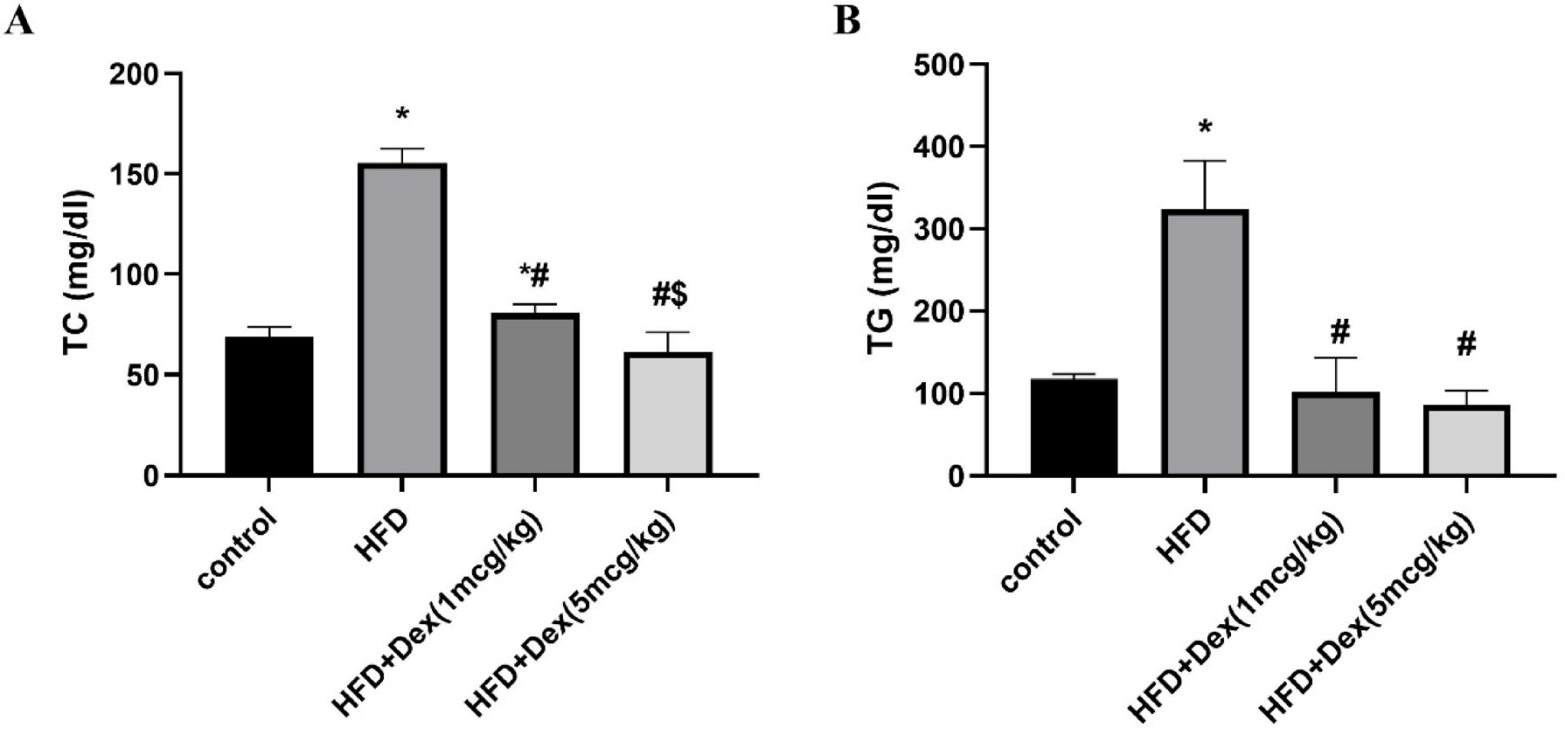
Plasma total cholesterol (TC) and triglycerides (TG) levels. Sprague Dawley rats were fed 60% HFD for 8 weeks, and Dex (1mcg/kg/day and 5mcg/kg/day) was given intraperitoneally. Lipid profile markers were assessed. Panels (**A**) and (**B**) correspond to TC and TG serum levels, respectively. Data are represented as mean ± SD (*n* = 6–8). ^*, #, $^*p* < 0.05, significantly different from Control, HFD, HFD + Dex (1mcg/kg), respectively, using one way ANOVA followed by Tukey post hoc test.

Hyperlipidemia, especially hypertriglyceridemia, is one of the hallmarks for HFD-induced MASLD. Therefore, lipid profile was assessed to ensure the establishment of the model and to assess the efficacy of Dex in alleviating hyperlipidemia. Compared to the control group, HFD increased TC and TG by 2.24-fold (from 69.1 ± 4.7 to 155.4 ± 7.2) and 2.76-fold (from 117.4 ± 5.6 to 323.7 ± 58.9), respectively. Dex (1 mcg/kg) and Dex (5 mcg/kg) caused significant reduction in TC levels by 48% (from 155.4 ± 7.2 to 80.5 ± 4.6) and 60% (from 155.4 ± 7.2 to 61.1 ± 9.9) (Fig. 3A) and TG levels by 68% (from 323.7 ± 58.9 to 102 ± 41.2) and 73.43% (from 323.7 ± 58.9 to 86 ± 17.6), respectively, Fig. 3B.

### Effect of Dex on oxidative stress markers MDA and GSH

**Fig. 4:**
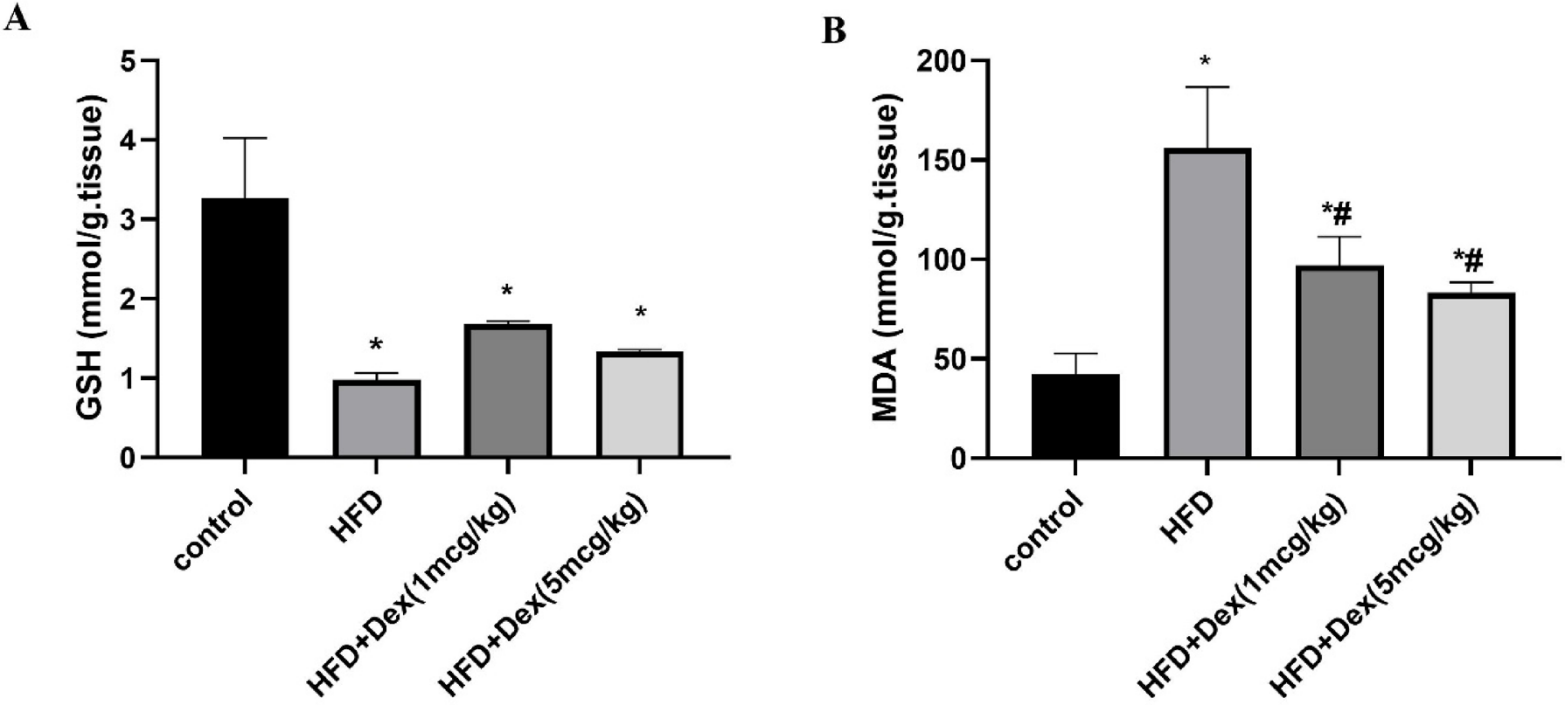
Oxidative stress markers. Sprague Dawley rats were fed 60% HFD for 8 weeks, and Dex (1mcg/kg/day and 5mcg/kg/day) was given intraperitoneally. oxidative stress markers file markers were assessed in liver homogenate. Panels (**A**) and (**B**) correspond to GSH, MDA levels, respectively. Data are represented as mean ± SD (*n* = 4). ^*, #^ *p* < 0.05, significantly different from Control, HFD, respectively, using one-way ANOVA followed by Tukey post hoc test.

Oxidative stress is one of the main consequences of HFD and it plays a crucial role in steatosis progression. In this study antioxidant marker GSH and the lipid peroxidation marker MDA were assessed in liver homogenate. Compared to the control group, HFD caused a reduction in GSH level by 69.9% (from 3.3 ± 0.8 to 0.98 ± 0.08) (Fig. 4A). HFD also caused an increase in MDA by 3.68-fold (from 42.4 ± 10.3 to 156 ± 30.7), Fig. 4B. Dex at (1 mcg/kg) and (5 mcg/kg) caused a marked increase in reduced GSH level by 1.7-fold (from 0.98 ± 0.08 to 1.7 ± 0.03) and 1.36-fold (from 0.98 ± 0.08 to 1.3 ± 0.02), respectively compared to the HFD group. On the other hand, HFD also resulted in the Dex at (1 mcg/kg) and (5 mcg/kg) resulted in reduction in MDA by 37.79% (from 156 ± 30.7 to 97 ± 14.3) and 46.6% (from 156 ± 30.7 to 83.3 ± 5.1), respectively, compared to HFD group.

### Effect of Dex on autophagic markers (AMPK, Beclin 1, ULK1 and LC3)

**Fig. 5:**
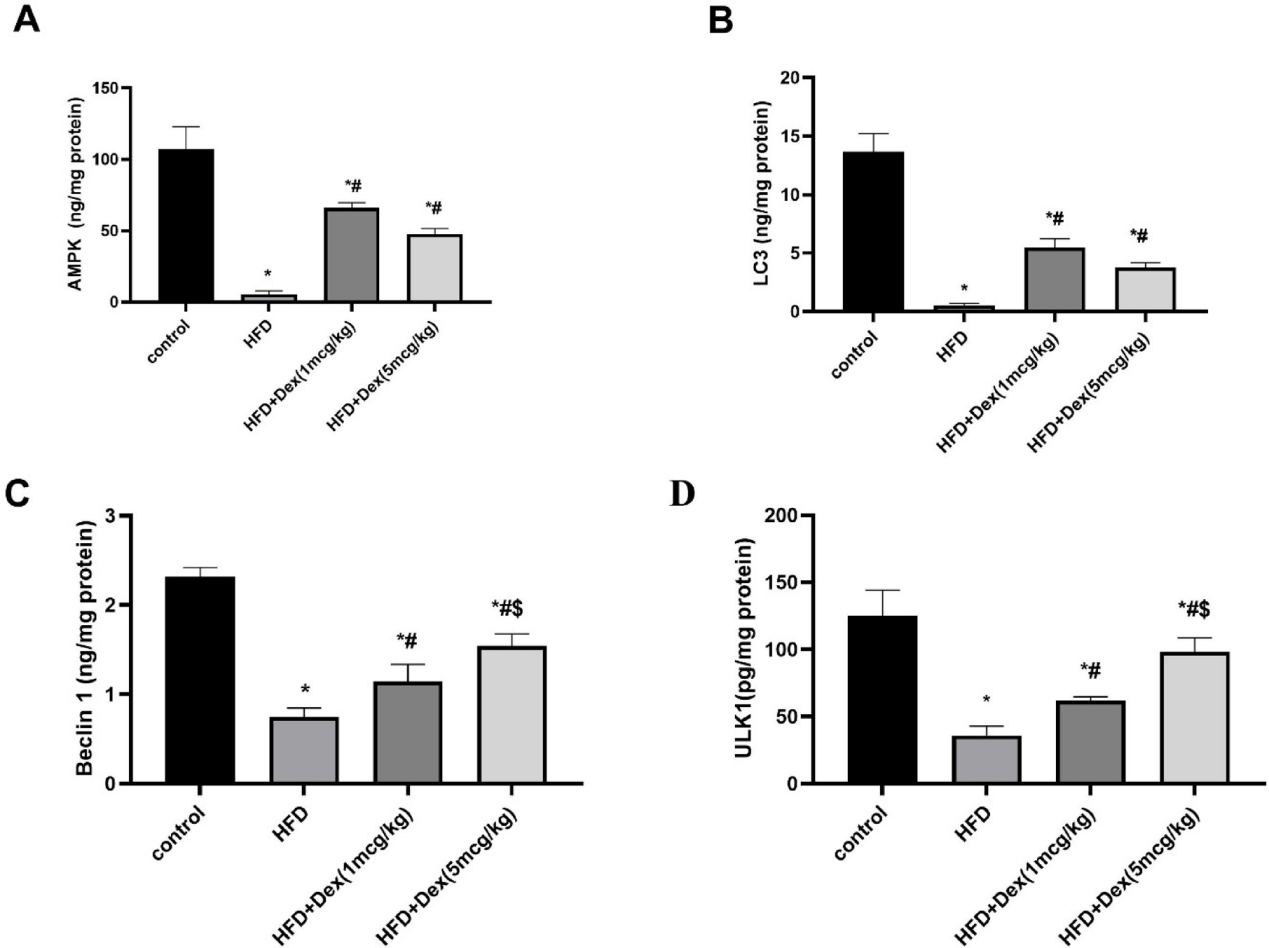
Autophagy markers levels. Sprague Dawley rats were fed 60% HFD for 8 weeks, and Dex (1mcg/kg/day and 5mcg/kg/day) was given intraperitoneally. Autophagic markers were assessed in liver homogenate. Panels (**A**), (**B**), (**C**) and (**D**) correspond to AMPK, LC3, Beclin1 and ULK1 levels, respectively. Data are represented as mean ± SD (*n* = 4). ^*, #, $^ *p* < 0.05, significantly different from Control, HFD, HFD + Dex (1mcg/kg), respectively, using one-way ANOVA followed by Tukey post hoc test.

To identify the mechanism by which Dex can protect against HFD, autophagy markers were assessed in liver homogenate. In HFD group, AMPK (Fig. 5A) and LC3 (Fig. 5B) decreased by 94.8% (from 107.1 ± 15.9 to 5.5 ± 2.4) and 96% (from 13.7 ± 1.6 to 0.5 ± 0.1), respectively, compared to control group. DEX (1mcg/kg) and DEX (5mcg/kg) groups caused a significant increase in AMPK by11.9-fold (from 5.5 ± 2.4 to 66.2 ± 3.6) and 8.6-fold (from 5.5 ± 2.4 to 47.6 ± 3.9) and in LC3 by 10-fold (from 0.5 ± 0.1 to 5.5 ± 0.7) and 6.9-fold (from 0.5 ± 0.1 to 3.8 ± 0.4), respectively. In HFD group Beclin1 (Fig. 5C) and ULK1 (Fig. 5D) decreased by 67.53% (from 2.3 ± 0.1 to 0.8 ± 0.1) and 71.3% (from 125 ± 19.2 to 35.9 ± 6.9), respectively. Dex (1mcg/kg) and Dex (5mcg/kg) groups caused a significant increase in Beclin1 by 1.52-fold (from 0.8 ± 0.1 to 1.1 ± 0.2) and 2.1-fold (from 0.8 ± 0.1 to 1.5 ± 0.1), respectively and in ULK1 by 1.72-fold (from 35.9 ± 6.9 to 61.8 ± 2.9) and 2.74-fold (from 35.9 ± 6.9 to 98.4 ± 10.2), respectively.

### Immunohistochemical assessment of p62

**Fig. 6:**
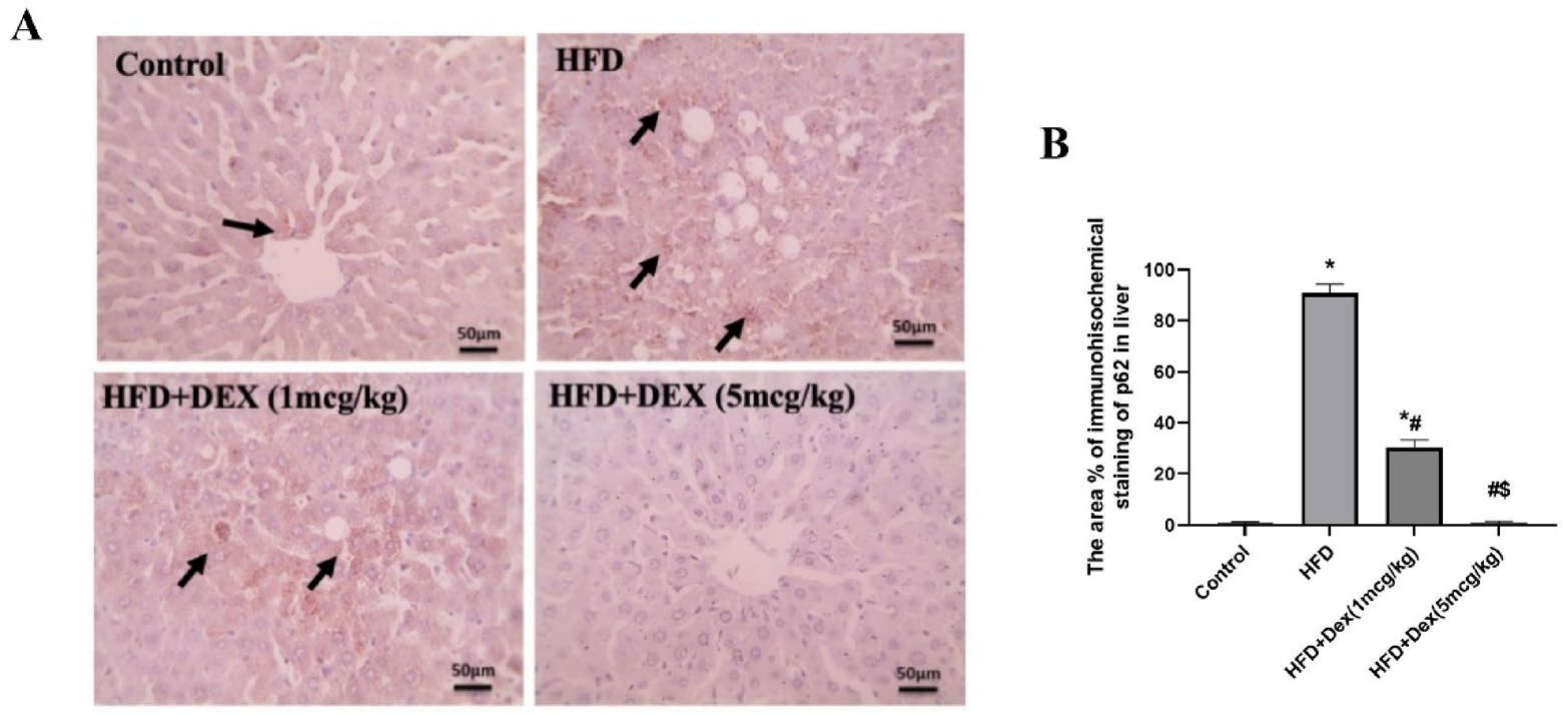
Representative IHC of p62 expression in hepatic sections from all experimental groups (**A**). Microscopic pictures of immunostained hepatic sections against p62 showing negative staining in hepatocytes in control group. Hepatic sections from HFD group showing marked positive brown cytoplasmic reaction that appears in many hepatocytes in areas of steatosis (black arrows). Hepatic sections from Dex-treated groups showing reduced positive brown cytoplasmic reaction that appears in a few hepatocytes in areas of steatosis (black arrows). Hepatic sections from Dex (1 mcg/kg) group show a very mild positive brown cytoplasmic reaction that appears in very few hepatocytes (black arrows). Hepatic sections from Dex (5 mcg/kg) group show a negative reaction in hepatocytes. Low magnification X: 100 bar 100 and high magnification X: 400 bar 50. IHC counterstained with Mayer’s hematoxylin. The area % of immunohistochemical staining of p62 is shown in (**B**). Data are expressed as mean ± SD (*n* = 6). ^*, #, $^ *p* < 0.05, significantly different from Control, HFD, HFD + DEX (1mcg/kg), respectively, using one-way ANOVA followed by Tukey multiple comparisons post hoc test.

To further investigate the effect of Dex on autophagy, p62 protein expression was evaluated. Microscopic pictures of immunostained hepatic sections against p62 showed negative staining in hepatocytes in control group. Hepatic sections from HFD group showed marked positive brown cytoplasmic reaction that appeared in many hepatocytes in areas of steatosis. The positive staining areas reduced gradually from Dex (1 mcg/kg) to Dex (5 mcg/kg), Fig. 6A. The area percentage of immunohistochemical staining of p62 is shown in Fig. 6B.

### Immunohistochemical assessment of apoptotic markers caspase-3 and Bcl-2

**Fig. 7:**
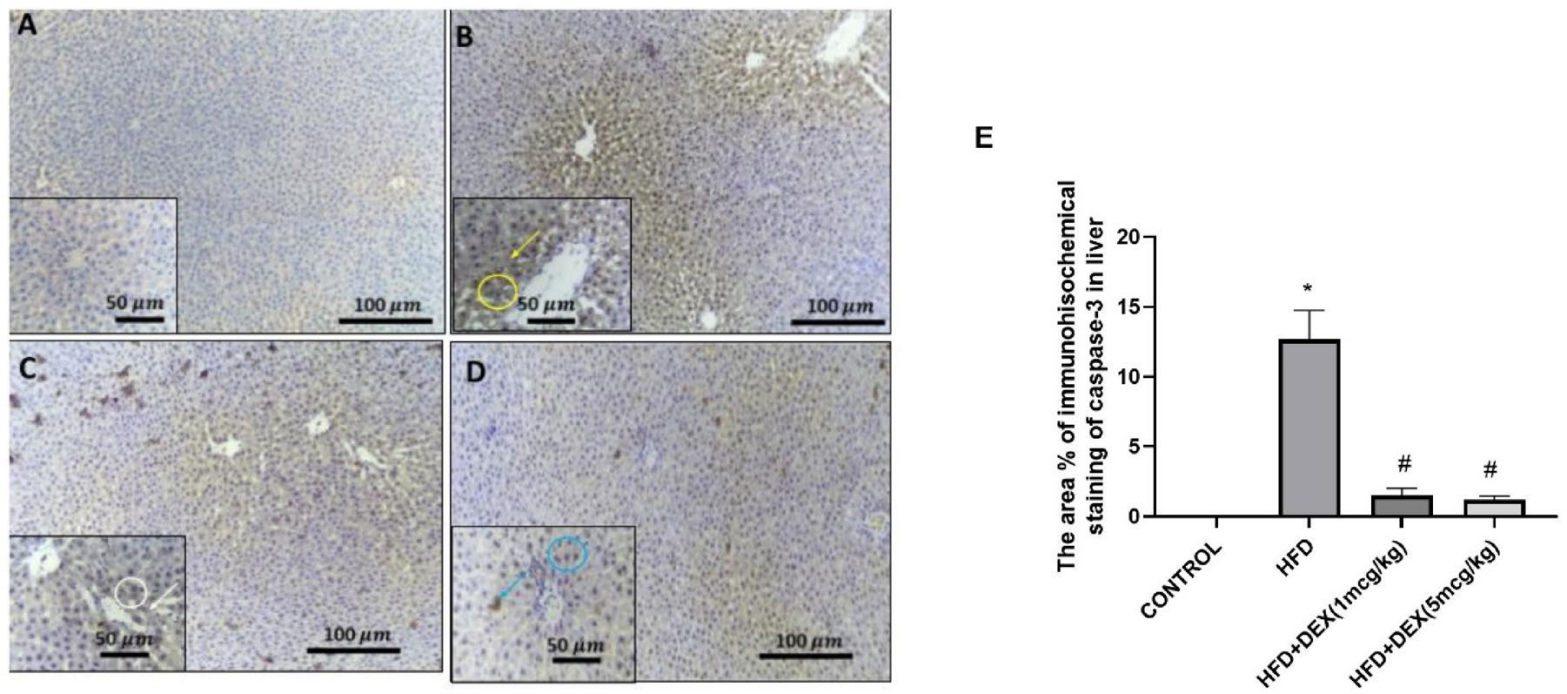
Representative IHC of Caspase-3 expression in hepatic sections. (**A**): Control group showing negative expression of caspase-3 in the hepatocytes. (**B**): HFD group showing diffuse immunopositively brown expression of Caspase-3 in the cytoplasm (yellow arrow) and nuclei (yellow circle) of hepatocytes. (**C**): Dex (1 mcg/kg) group showing mild faint brown immunopositively cytoplasmic (white arrow) and nuclear (white circle) expression of Caspase-3 in the hepatocytes. (**D**): Dex (5 mcg/kg) group showing minimal to mild nuclear (blue circle) and cytoplasmic (blue arrow) expression of Caspase-3. Image magnifications, X:100, Bar=100 μm, the in site: X:400, Bar=50 μm. The area percentage of immunohistochemical staining of Capase-3 is shown in (**E**). Data are expressed as mean ± SD (*n* = 3). ^*, #, $^ *p* < 0.05, significantly different from Control, HFD, HFD + DEX (1mcg/kg), respectively, using one-way ANOVA followed by Tukey multiple comparisons post hoc test.

To investigate the effect of Dex on apoptosis, caspase-3 and Bcl-2 protein expression were evaluated. Microscopic pictures of immunostained hepatic sections against caspase-3 showed negative staining in hepatocytes in control group, Fig. 7A. Hepatic sections from HFD group showed diffuse immunopositive brown expression of Caspase-3 in the cytoplasm and nuclei of hepatocytes, Fig. 7B. Dex (1 mcg/kg) showed mild, faint brown immunopositively cytoplasmic and nuclear expression of Caspase-3 in the hepatocytes, Fig. 7C and Dex (5 mcg/kg) showed minimal to mild nuclear) and cytoplasmic expression of Caspase-3, Fig. 7D.

**Fig. 8:**
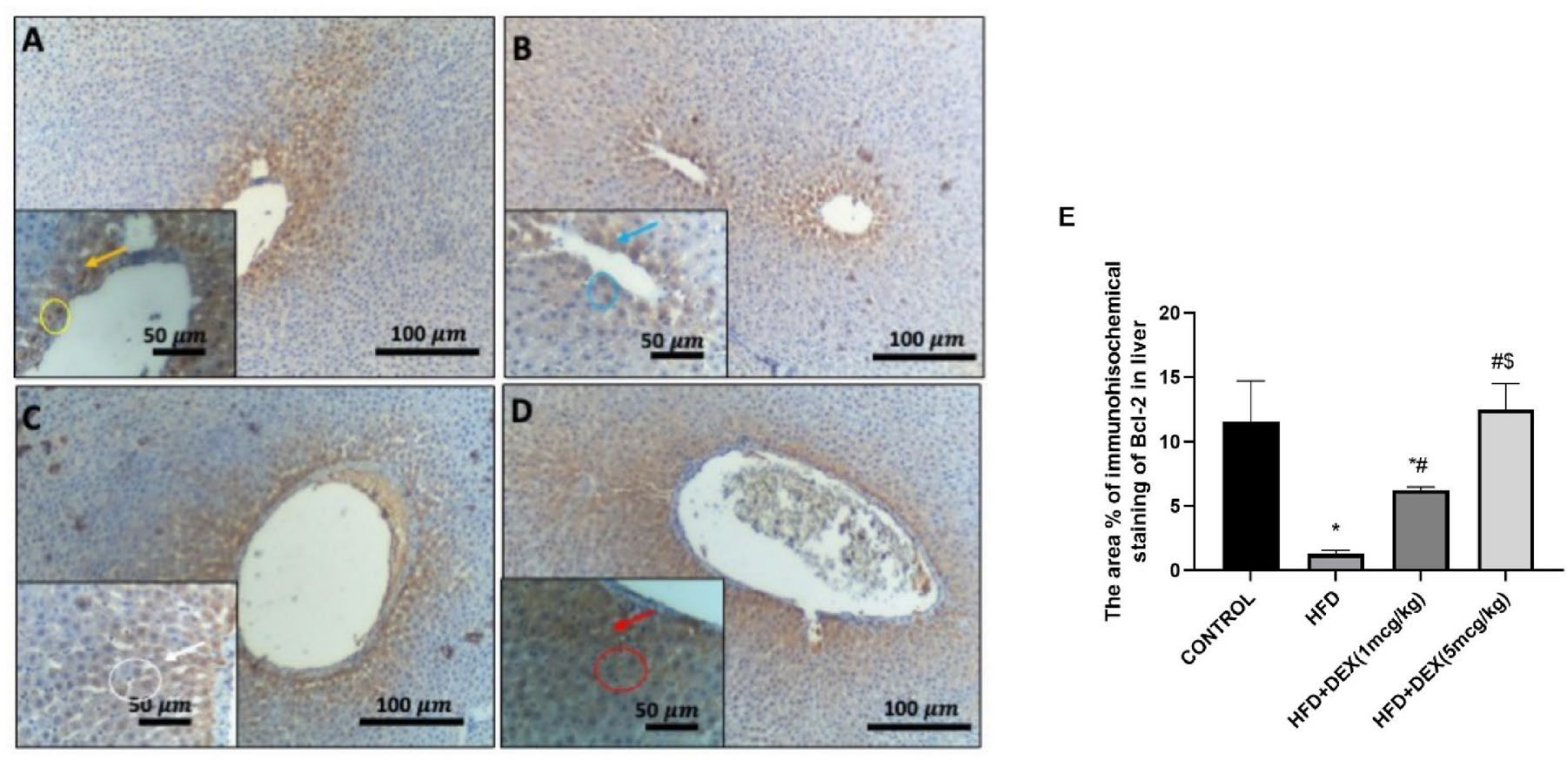
Representative IHC of Bcl-2 expression in hepatic sections. (**A**): Control group showing diffuse intense nuclear (yellow circle) and cytoplasmic (yellow arrow) expression of the Bcl-2 in the hepatocytes. (**B**): HFD group showing minimal to mild immunopositively brown expression of Bcl-2 in the cytoplasm (blue arrows) and nuclei (blue circles) of hepatocytes. (**C**): HFD + Dex (1mcg/kg) group showing mild to moderate immunopositively brown expression (white arrow) and nuclear (white circle) expression of Bcl-2 in the hepatocytes. (**D**): HFD + Dex (5mcg/kg) group showing diffuse, intense immunopositively stained hepatocytes (orange arrow). Image magnifications, X:100, Bar=100 μm, the in site: X:400, Bar=50 μm. The area percentage of immunohistochemical staining of Bcl-2 is shown in (**E**). Data are expressed as mean ± SD (*n* = 3). ^*, #, $^ *p* < 0.05, significantly different from Control, HFD, HFD + DEX (1mcg/kg), respectively, using one-way ANOVA followed by Tukey multiple comparisons post hoc test.

Microscopic pictures of immunostained hepatic sections against Bcl-2 showed diffuse intense nuclear and cytoplasmic expression of the Bcl-2 in the hepatocytes in control group, Fig. 8A. Hepatic sections from HFD group showed minimal to mild immunopositively brown expression of Bcl-2 in the cytoplasm and nuclei) of hepatocytes, Fig. 8B. The positive staining areas increased gradually from Dex (1 mcg/kg) to Dex (5 mcg/kg), Fig. 8C and D.

The area percentage of immunohistochemical staining of Capase-3 was significantly increased in HFD group as compared to the control group. Meanwhile, both doses of Dex managed to decrease the protein expression of Caspase-3, as shown in Fig. 7E. In addition, the area percentage of immunohistochemical staining of Bcl-2 was significantly decreased in HFD group as compared to the control group. In contrast, both doses of Dex managed to increase the protein expression of Bcl-2 as shown in Fig. 8E.

## Discussion

Diets containing more than 30% of energy from fat are considered HFDs and they are used as models of diet-induced obesity (DIO) and liver steatosis^28^.

MASLD is a leading cause of chronic liver disease around the world. MASLD is a disease spectrum characterized by hepatic steatosis when no other causes of secondary hepatic fat accumulation can be detected (e.g., excessive alcohol consumption). MASLD spans from the more benign condition of steatotic liver to the more severe condition of MASH. MASLD can lead to fibrosis and cirrhosis^29,30^. So far, the pathognomonic lesion of steatosis has been associated with fat buildup in specialized subcellular organelles known as lipid droplets (LDs). LD catabolism was formerly assigned solely to cytosolic lipases, although lipophagy has recently been described as an additional mechanism for hepatic LD mobilization^8,31^. Dysfunction of lipophagy has been closely linked to MASLD pathogenesis^32,33^.

Lipophagy consists of the following steps: (1) metabolic upstream regulation of lipophagy inducers; (2) protein-mediated sequestration of cargo (a portion or the entire LD) within the phagophore, a cytosolic double-membrane vesicle, and subsequent formation of the autophagosome via various mechanisms, including chaperone-mediated autophagy (CMA), microautophagy, and macroautophagy^34^. (3) autophagosome tracking and delivery to the lysosome, as well as their fusion; and (4) cargo breakdown by lysosomal lipases. All of these stages might be compromised at varying levels, and new evidence links autophagy dysfunction to MASLD^35,36^. Each event of the autophagic process is coordinated by a complex network of more than 32 genes and their protein products (autophagy-related genes (ATGs) and proteins (Atgs)^37^.

Dex is a highly selective agonist of the adrenergic 2 receptor having sedative, analgesic, anti-inflammatory, and anti-sympathetic properties^38^. Furthermore, numerous experimental research employing diverse animal models have indicated that Dex protects against multiple organ damage^19^.

In a previous study, it was proved that Dex Ameliorated Early Liver dysfunction, decreased the levels of inflammatory cytokines in septic mice, induced autophagy by upregulating the SIRT1/AMPK pathway, which is a key survival-signaling pathway involved in modulating autophagy, increased the expression of beclin-1, LC3II and decreased the expression of p62^18^.

In the present study, ALT and AST were elevated in HFD group compared to control group. Elevated blood aminotransferase levels may be linked to liver damage. Testing for AST or ALT is part of many routine screening approaches^39^. Dex treatment reduced the degree of hepatocellular damage evidenced by decreased serum ALT and AST levels. GGT is an important transferase that participates in the transpeptidation of functional gamma-glutamyl groups to different receptor moieties. It plays critical roles in antioxidant defense processes. In addition, GGT is very sensitive for the diagnosis of liver injury and metabolic syndrome^40^. In this study, GGT increased in HFD group compared to control group; meanwhile, Dex treatment reduced GGT levels.

As previously stated, MASLD is related with oxidative stress and the formation of reactive oxygen species (ROS). We assessed GSH as an antioxidant defensive marker and MDA as a reactive carbon molecule and lipid peroxidation marker in our study. MDA increased in HFD group compared to control group. Dex treatment reduced MDA levels. GSH decreased in HFD group and increased in Dex groups, however these modest increases were not statistically significant.

Beclin 1 refers to a core component of Class III phosphatidylinositol 3-kinase (PI3K-III) complex. This complex is involved in membrane transport and remodeling during autophagy^41^. Beclin 1 regulates the lipid kinase activity of the PI3K-III catalytic unit VPS34, which is responsible for the synthesis of phosphatidylinositol 3-phosphate (PI3P). As a result, multiple autophagy proteins involved in autophagosome nucleation are recruited. Beclin 1 functions as an adapter, attracting various proteins that modulate VPS34. Beclin 1 deficiency destabilizes the Class III PI3K complex, impairing VPS34 activity, autophagic flux, and endocytic trafficking^42,43^. Dex treatment increased autophagy induction evidenced by increasing beclin 1 protein levels.

In mammals, UNC-51-like kinase 1 (ULK1), a serine/threonine kinase, is an autophagic initiator. ULK1 is well-known for activating autophagy, which is evolutionarily conserved in protein transport and required for cell homeostasis^44^. In our study we used ULK1 as a marker for autophagy induction. In Dex groups, ULK1 was significantly increased compared to HFD group.

In animals, LC3-II, a lipidated variant of LC3, has been proven to be an autophagosomal marker^45^. LC3-II modulates the elongation of phagophore membranes^46^ and facilitates membrane tethering and hemifusion^47^. Autophagic induction by Dex was seen as a significant increase in LC3-II when compared to HFD group.

The autophagic-lysosome pathway degrades p62, an autophagy adaptor protein. The accumulation of p62 protein is caused by a lack of autophagy^48^. In our study, immunohistochemical analysis of p62 showed a marked increase of p62 in HFD groups compared to control group and a marked decrease in Dex groups, proving autophagic induction.

AMPK modulates autophagy at various stages. First, AMPK directly phosphorylates the mTOR upstream regulator TSC2 at Thr1227 and Ser1345^49^. as well as the mTORC1 component RAPTOR at Ser722 and Ser792^50^. Both of these phosphorylation events contribute to the reduction of mTOR activity in response to energy stress. Reduced mTOR activity relieves the inhibitory phosphorylation of ULK1 and activates autophagy. Second, the discovery that AMPK directly phosphorylates ULK1 established a direct link between AMPK and the primary autophagy process^51^. In our study, we used AMPK as a marker for autophagy induction. In DEX groups, AMPK was significantly increased compared to HFD group.

The relationship between autophagy and cell death has been studied, and many experts believe the two processes are related^52^. Both extrinsic and intrinsic mechanisms are typically involved in cellular apoptosis. The extrinsic route is governed by death receptors in the cell membrane, which initiate the caspase cascade. The intrinsic pathway, on the other hand, is strongly tied to the Bcl-2 family of proteins, which all regulate apoptosis by controlling the permeability of the mitochondrial outer membrane^53,54^. In our study, immunohistochemical analysis of caspase-3 and Bcl-2 showed a significant increase of caspase-3 and decrease of Bcl-2 in HFD groups compared to control group and a significant decrease of caspase-3 and increase of Bcl-2 in Dex groups in a dose-dependent manner, proving a significant inhibition of apoptosis.

## Conclusion

Dex treatment induces the autophagic pathway in the liver, especially macroautophagy (lipophagy) in MASLD in a dose-dependent manner, improved liver functions and degree of steatosis. Therefore, Dex may be a potential adjunctive therapeutic option in clinical settings of treating MASLD.

## Limitations to this study

In the current study, Dex was given concurrently with the HFD. Future studies should focus on the role of Dex as a treatment option in an already steatotic liver. In addition, only male rats were used in our study, therefore, future studies should be carried out using both female and male rats to study the effect of sex polymorphism as the disease progression can vary between the sexes.

## Data Availability

The data are available from the corresponding author on reasonable request.
